# Ketogenic Weight Loss Diet Rapidly Unmasks an Insulinoma

**DOI:** 10.1155/2019/9674245

**Published:** 2019-10-27

**Authors:** Erin Meier, Lydia Winnicka, Michael Lau, Duc Vo, Alla Joutovsky, Mangalore Amith Shenoy

**Affiliations:** ^1^Department of Internal Medicine, NYU Winthrop, USA; ^2^Department of Pulmonary and Critical Care Medicine, NYU Winthrop, USA; ^3^Department of Pathology and Laboratory Medicine, NYU Winthrop, USA

## Abstract

The ketogenic diet, a diet high in fat and extremely low in carbohydrates, has recently gained momentum and is increasingly employed by patients in order to lose weight. We report a case of an otherwise healthy 47-year-old male who developed seizures and hypoglycemia shortly after initiating this diet. Biochemical testing confirmed hyperinsulinemic hypoglycemia and a subsequent abdomen MRI showed a pancreatic mass. The tumor was ultimately excised with pathology confirming an insulinoma. It was felt that his abrupt decrease in carbohydrate consumption led to the rapid unmasking of this tumor.

## 1. Introduction

The ketogenic diet is increasingly employed by the general public in order to lose weight. A standard ketogenic diet is typically very high in fat, with 65%–75% of calories coming from fat, and extremely low in carbohydrates, with 5%–10% of calories coming from carbohydrates. By significantly restricting carbohydrate intake, blood glucose and insulin levels drop, and the body's response is to use an alternate fuel source, namely fat, which can then lead to desired weight loss. Insulinomas are rare pancreatic tumors that pathologically secrete insulin and can cause significant hypoglycemia. We present a case of a 47-year-old male who began the ketogenic diet and within 1 week, developed severe hypoglycemia with eventual diagnosis of an insulinoma.

## 2. Case Report

**Figure 1:**
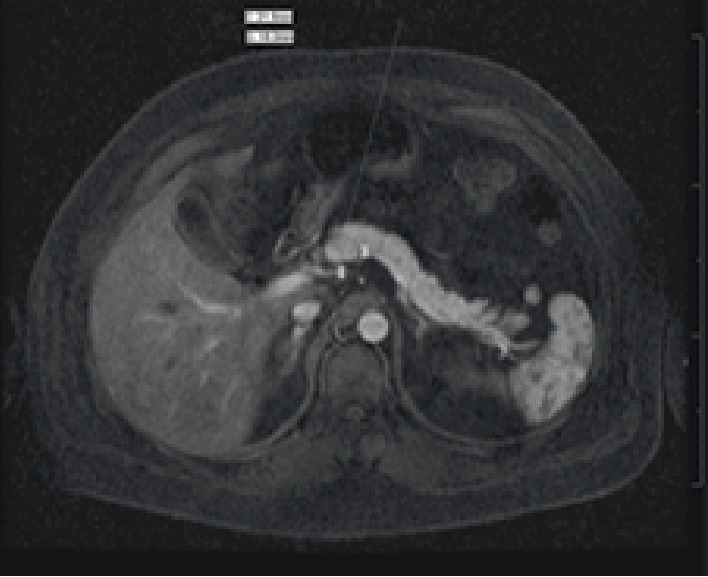
Magnetic resonance imaging T1 early arterial phase with arrow indicating mass.

A 47-year-old Hispanic male, with a BMI of 38 and no past medical history, presented to the Emergency Department (ED) with seizure activity. One-week prior, he had initiated a ketogenic diet in an attempt to lose weight. Prior to that, he did not follow any specific diet. He began predominantly consuming red meat, dairy, and eggs, and completely cut out grains, fruits, soda and juice. His goal was to consume as few carbohydrates as possible. During this time, he began to feel more weak and tired, but otherwise had no specific complaints. On the day of the presentation, he felt weaker and lay down to rest. His wife then observed him to become unresponsive and have rhythmic jerking movements, consistent with seizure activity. On arrival to the ED, he was lethargic but had normal vital signs and was saturating well on room air. He was found to have a glucose level of 42 mg/dL. His symptoms quickly improved with dextrose. Insulin level on presentation was 87.9 uIU/mL, with a c-peptide level of 8.9 ng/mL and a beta-hydroxybutyrate level of 0.06 mmol/L. Insulin antibody was negative. Given there was concern for an insulinoma, he underwent a prolonged fasting test, which confirmed the diagnosis of hyperinsulinemic hypoglycemia. Two hours into fasting, he felt lightheaded and was found to have a glucose level of 38 mg/dL, however, he had received intravenous dextrose two hours before. As it was difficult to maintain safe serum glucose levels whenever the dextrose infusion was stopped, he received multiple doses of diazoxide. Twenty-four hours after the dextrose infusion was stopped and the last dose of diazoxide was given, he began a second prolonged fasting test. Five hours after initiation, he became symptomatic with a glucose level of 56 mg/dL and a corresponding insulin level of 16.1 uIu/mL. Whipple's triad was documented at this time. Subsequently, a contrast-enhanced MRI of the abdomen and pelvis was done and revealed a 2.2 × 1.5 cm mass in the pancreatic neck/body, compatible with a neuroendocrine tumor (Figure 1).

**Figure 2:**
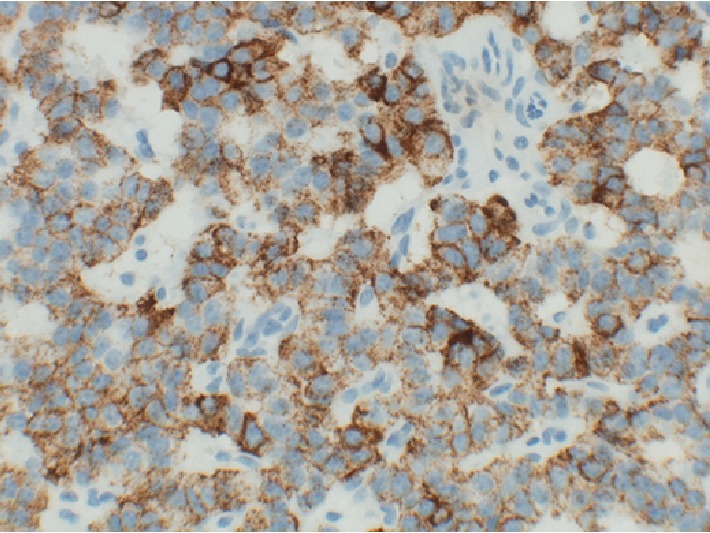
Neoplastic cells with positive cytoplasm stain for insulin, indicating an insulinoma.

Given prior biochemical testing, an insulinoma was felt very likely. Surgical enucleation of the mass was performed during hospital admission. Gross pathology revealed a 2 × 1.7 × 1.5 cm encapsulated mass with central hemorrhage. Light microscopy showed neoplastic cells with a trabecular architecture and a salt-and-pepper nuclear pattern, typical of neuroendocrine tumors. The neoplastic cells had a positive cytoplasmic stain for insulin (Figure 2).

In the post-operative period, he had no further episodes of hypoglycemia. Glucose levels remained between 70 and 120 mg/dL while in the hospital. No post-operative insulin level was obtained. At two months follow up, he again remained asymptomatic with no episodes of hypoglycemia.

## 3. Discussion

There has recently been increased public interest in a ketogenic diet as a means of weight loss. Originally proposed as a therapy for epilepsy, ketogenic diets have since been used in order to maintain or lose weight. Currently, the Obesity Medicine Association endorses a low or very-low carbohydrate diet as one option for weight loss [1]. The main tenant of this diet is that when carbohydrates are severely restricted, the body will enter a state of ketosis and will break down fat as a fuel source, which will lead to weight loss. In order to enter ketosis, blood glucose levels must decrease and glycogen stores must be depleted [2]. In the absence of glucose to trigger the release of insulin, insulin levels should then decrease. This combination will force the body into ketogenesis, which will convert fatty acids into ketones, namely acetate, acetoacetate, and beta-hydroxybutyrate, to use as an alternate fuel source. While on a strict ketogenic diet, insulin levels should be very low. Our patient had a pathological condition where his insulin levels were actually very elevated. As insulin halts the utilization of fat as fuel, he developed hypoglycemia abruptly upon eliminating carbohydrates from his diet. He was thus unable to utilize either glucose or fat as an energy source [3]. His beta-hydroxybutyrate level on admission was normal, indicating he had not been able to reach a state of ketosis, due to pathologically high insulin levels. Prior to initiating this diet, he did not have any apparent episodes of symptomatic hypoglycemia. He had presumably never restricted his carbohydrate intake before this point though.

Insulinomas are rare, insulin-secreting pancreatic tumors, with an incidence of approximately 1–4 per one million [4]. Though the biochemical diagnosis of an insulinoma is straightforward, there is a median duration of symptoms prior to diagnosis of 1.5 years, due to the often nonspecific presentation [5]. The symptoms of insulinomas, namely that of hypoglycemia, include confusion, diaphoresis, palpitations, and seizures. These symptoms may be attributed to other causes initially, and can lead to a delay in diagnosis. Once an insulinoma is considered, diagnostic criteria includes a prolonged fasting test, followed by imaging to localize the tumor [4]. The prolonged fasting test can detect up to 99% of insulinomas and must be performed in a controlled setting such as an intensive care unit [6]. Insulin levels, C-peptide levels, and glucose levels are measured once the patient experiences symptoms of hypoglycemia. Unfortunately, the first prolonged fasting test that our patient underwent was complicated by the fact that he had received intravenous dextrose within two hours of symptom onset. As reactive hyperinsulinemia resulting in hypoglycemia could not be ruled out, it was necessary to give diazoxide to our patient to inhibit insulin release, in order to allow discontinuation of any dextrose infusion. Diazoxide, which has both a hyperglycemic effect and lowers insulin levels, is commonly used as medical therapy in patients who are not surgical candidates or who refuse surgery. Other medical options include somatostatin analogs, which at high doses inhibit the secretion of insulin [7]. Our patient's second prolonged fasting test ended with a blood glucose of 56 mg/dL and insulin level of 16.1 uIU/mL. Although an insulin level of 16 uIU/mL can fall within the normal range, a glucose level of less than 50 mg/dL should result in a much lower insulin level, around 6 uIU/mL [7].

It is unclear how long our patient had an insulinoma, or when it would have been discovered, had he not started the ketogenic diet. Our patient did not retrospectively identify any symptoms of hypoglycemia, as most patients with insulinomas do. We believe his decision to begin the ketogenic diet directly led to the rapid diagnosis of his tumor. Understanding the physiology of the ketogenic diet led us to quickly suspect an insulinoma as the most likely diagnosis. Our case is the first to our knowledge of a ketogenic diet rapidly unmasking an insulinoma and illustrates the importance of taking a thorough history and understanding the physiologic implications of different popular diets.

## 4. Conclusion

The premise of the ketogenic diet is that with low carbohydrate intake, the body will convert fat into fatty acids and use ketone bodies as energy, which can lead to weight loss. However, if insulin levels are high, then fat cannot be converted into energy and the body will not be able to reach a state of ketosis. After beginning the ketogenic diet, due to the presence of an insulinoma, our patient was unable to utilize either glucose or fat as energy. He therefore quickly developed symptomatic hypoglycemia and presented to the ED with seizures. Though hypoglycemia can occur in all persons on carbohydrate-restricted diets, the ketogenic diet has a much greater carbohydrate restriction, and therefore more often leads to hypoglycemia. This, coupled with a previously undiagnosed insulinoma, lead to rapid symptom unmasking. Understanding the physiology of the ketogenic diet helped to make a prompt diagnosis of an insulinoma in our patient.
